# Direct Medical Cost of Treating Substance Use Disorders in Two Tertiary Hospitals in South-West, Nigeria: A Cross-Sectional Study

**DOI:** 10.1155/2022/6320141

**Published:** 2022-01-15

**Authors:** Margaret Ilomuanya, Ogochukwu Amaeze, Chinenye Umeche, Ugochukwu Mbata, Omonike Shonekan, Abayomi Olajide

**Affiliations:** ^1^Department of Pharmaceutics and Pharmaceutical Technology, Faculty of Pharmacy, University of Lagos, Lagos, Nigeria; ^2^Department of Clinical Pharmacy and Biopharmacy, Faculty of Pharmacy, University of Lagos, Lagos, Nigeria; ^3^Federal Neuro-Psychiatric Hospital, Yaba, Lagos, Nigeria; ^4^Department of Mathematics and Statistics, Faculty of Science, University of Lagos, Nigeria; ^5^Department of Pharmaceutical Chemistry, Faculty of Pharmacy, University of Lagos, Lagos, Nigeria; ^6^Federal Neuro-Psychiatric Hospital, Aro Abeokuta, Ogun State, Nigeria

## Abstract

**Introduction:**

Successful interventions for substance use disorders (SUDs), though obtainable, are not effectively utilized due to the high cost of treatment. The adoption of any given therapy is often impeded by insufficient evidence of the effectiveness of such treatment.

**Objective:**

This study aimed to assess the direct medical cost of treating SUD in two tertiary hospitals in South-West, Nigeria.

**Methods:**

A descriptive, cross-sectional survey of patients managed for SUD at the two psychiatric hospitals was carried out between January and June 2020. The inclusion criteria were patients with SUD above 18 years of age, registered and managed at the two hospitals. Data were collected from selected patients' case notes using a standardized data collection tool and analyzed using descriptive and inferential statistics.

**Results:**

The average costs of treatment for alcohol use disorder, drug use disorder, and drug and alcohol use disorder were ₦146,425.38 ± 57,388.84, ₦135,282.09 ± 53,190.39, and ₦143,877.33 ± 68,662.04, respectively. This translates to $384.82, $355.53, and $378.12, respectively. The highest contributors to SUD treatment cost are inpatient admissions and the cost of medicines; inpatient admissions include accommodation, feeding, and laundry.

**Conclusion:**

Considering that over 60% of the Nigerian population lives below the poverty line, the direct cost of SUD treatment is unaffordable to the patients and the health care system, which is grossly underfunded.

## 1. Introduction

Although available to patients in Sub-Saharan Africa, interventions for substance misuse disorders are usually dependent on the patient's ability to bear the associated costs [1–3]. Utilization of specific therapy for the management of substance abuse is generally based on empirical evidence of effectiveness. However, scarce resources in hospital settings mitigate against the adoption of particular treatments [4].

Over 30 million individuals worldwide are managed for substance abuse, with 50% explicitly being managed for opioid abuse. Of this number, only a 10th of the patients are currently in therapy despite the obvious sociological and psychosocial consequences of not accessing treatment [5]. It costs northern American governments over $90 billion annually in economic costs to currently manage opioid abuse. An estimated 14.4% of the Nigerian population has used psychoactive drugs recreationally; this includes individuals who have been termed, high-risk drug users [6]. Users of poly-drugs, such as diacetylmorphine (heroin), 2-[(Dimethylamino) methyl]-1-(3-methoxyphenyl) cyclohexanol (tramadol), benzoylmethylecgonine (cocaine), and morphine, account towards the statistics of substance abusers [7]. In Nigeria, cannabis, commonly known as marijuana, is the most commonly abused substance. Codeine and codeine-containing orally used preparations also account for over 20% of active opioid drug abusers in Nigeria. The use of injectable opioids has also been observed in most high-risk users. High-risk users in Nigeria were estimated at 376,000 adults 15 to 64 years old between 2017 and 2019 [8, 9].

In Nigeria, substance use disorder (SUD) patients are usually managed using a continuum of care that comprises early intervention using pharmacological and nonpharmacological approaches to prevent more severe substance use disorders. This is generally achieved via hospitalization in neuropsychiatric hospitals. SUD patients are typically admitted through the emergency unit of the hospital. A detailed history of drug use is taken at admission, and family history, developmental, forensic and psychosocial histories, and other relevant data are collected. A positive urine test for psychoactive substance(s) is a prerequisite for admission, and in some cases, involuntary admission usually mandated by an authority is acceptable.

The SUD treatments offered at the hospitals include psychotherapy interventions (such as cognitive behavioral therapy, individual and group counseling), pharmacotherapy to treat co-occurring disorders (medical or mental), and to aid in acute withdrawal symptoms, occupational skills training, relapse prevention training, skills development training, family therapy, motivational enhancement therapy, and motivational interviewing. In addition, psychosocial support through religious and social group networks is leveraged during counseling according to the patients' belief system. It can thus be said that a multiprong approach is utilized in managing drug-addicted patients in Nigeria.

Despite the perception that SUD poses a significant public health problem in Nigeria, little information about its economic burden exists. An economic evaluation will bolster the health care system with veritable data to ensure appropriate funds are provided along the cascade of primary, secondary, and tertiary health care facilities to tackle this SUD. This study details the demographics, medical resources utilized, and the average cost of treatment across the patients' demographic characteristics. These data are relevant for health policy and practice decisions to stimulate increased budgetary allocation amidst the lack of health funds to the SUD management. The objective of this study was to assess and determine the drivers of the direct medical costs of treating patients with substance use disorder in two tertiary hospitals in South-West, Nigeria, while also postulating the affordability of such cost to the patients. This assessment will be critical to providing veritable information for health care systems in Nigeria to ensure health planning in both private and public sectors.

## 2. Materials and Methods

This study's perspective is that of the patients. Therefore, it attempts to assess the direct costs of treating substance use disorder and its related complications on patients. To explore these costs per patient, we evaluated the treatment profiles of a representative sample of patients managed for substance use disorder for 6 months.

### 2.1. Study Design, Period, and Setting

The research was a descriptive, cross-sectional survey of clients managed for SUDs at two psychiatric hospitals between January and June 2020. The inclusion criteria were patients with any type of SUD who are above 18 years of age, registered, and managed at the two hospitals. Patients who were not managed for SUD and pregnant patients were excluded from the study. Both inpatients and outpatients were recruited into the study.

This study was carried out at two psychiatric hospitals in South-West, Nigeria, namely, Federal Neuro-Psychiatric Hospital (FNPH), Lagos, and Neuropsychiatric Hospital, Aro, Abeokuta, Ogun state. Both hospitals are tertiary health care facilities attending to the states' mental health care needs. FNPH is a 535-bed hospital located at Yaba, a suburb in Lagos mainland. The hospital was set up as a lunatic asylum under the British Colonial Mile, transformed into a full psychiatric hospital in 1977. The hospital has an annex at Oshodi that deals mainly with children and adolescents with mental illnesses. The Neuropsychiatric Hospital, Aro, Abeokuta, Nigeria, started as an asylum at Lantoro, Abeokuta, in 1944. In 1954, the psychiatric hospital came into existence to care for mental and nervous diseases, with the antecedent, Lantoro as an annex of the hospital and currently has a bed space of 537.

### 2.2. Sample Size Calculation and Sampling

The sample size was calculated using Cochran's formula:

(1) $$\begin{array}{c} N_{o}=\frac{Z^{2}Pq}{e^{2}}, \end{array}$$

where *Z* is the statistic corresponding to 95% level of confidence = 1.96; *P* is the expected prevalence obtained from same studies or a pilot study; and *d* is the margin of error or precision. Using a prevalence of SUD treatment as determined from previous studies as 11.6% [10], a 95% confidence interval (*Z* = 1.96), and a prevalence estimate within 5% error margin (*d*), a minimum of 158 samples was calculated and deemed appropriate for this study.

### 2.3. Data Collection Instrument and Techniques

Data were obtained from the patients' case notes retrieved from the respective study sites' records departments. Case notes of all eligible for the study were selected, and the ones used for the study were randomly selected from those case notes. Data were obtained with the aid of a data collection tool that was pretested in a pilot study of 10 randomly selected patients' case notes to ensure the tool's feasibility and suitability to achieve the study's intended objectives. The data collected included the patients' demographics, costs of prescribed medicines, laboratory tests, consultation fees, psychosocial/behavioral therapy, inpatient admission, and medical devices. These data were meticulously extracted from the selected patients' case notes by intern pharmacists trained on such data collection. Direct health care costs were calculated based on the patients' payments for medicines, inpatient admissions, laboratory tests, behavioral or cognitive therapy, use of medical devices, and consultation fees. The costs of all the items for each patient during the 6-month study period were summed up to obtain the total direct treatment costs. These costs were then averaged over all patients in each SUD category to get the average cost. The SUD categories are alcohol use disorder (AUD) and drug use disorder (DUD).

### 2.4. Data and Statistical Analysis

The collected data were checked for completeness; responses were coded and inputted into IBM SPSS version 21.0 for Windows for statistical analyses. Descriptive statistics using frequencies and percentages were used to evaluate the patients' socio-demographic characteristics. Pearson's chi-square test was used to test for association between the patients' socio-demographic characteristics and SUDs. Student's *t*-test and *F*-test were used to test for significant differences in socio-demographic characteristics among the two categories of SUD (*p* < 0.05). One-way analysis of variance (ANOVA) was used to test for significant variation among the different categories of SUDs on the associated direct cost of treatments.

## 3. Results

### 3.1. Socio-Demographic Characteristics of the Patients

A total of 199 patients' case notes were assessed: 162 (81.4%) from Federal Neuro-Psychiatry hospital, Lagos state, and 32 (18.6%) from Neuropsychiatry Hospital, Aro, Abeokuta, Ogun state, Nigeria. The study population had a median (interquartile range) age of 23.06 (5–63.5) years, and the 20 to 29 years age group had the highest number of patients (72 [40.2%]). There were more males (80.90%) than females (19.1%), and 167 (83.92%) of this population were unmarried. One hundred and twenty-seven (63.81%) of the patients had attained tertiary education, while 66 (33.16%) had secondary education. Regarding occupation, students (32.67%) and unemployed persons (36.18%) were the most recorded amongst the study population (Table 1). Pearson's chi-square test showed an association between age, marital status, occupation, and patient's state of residence and substance use disorders (AUD, DUD, or both) at chi-square values = 18.612, 17.492, 23.330, and 36.662, respectively; *p* < 0.05 (Table 1).

### 3.2. Clinical History of the Patients

Almost half of the study population [91 (45.73%)] were managed for DUD, 86 (43.21%) were managed for AUD, while 22 (11.05%) were managed for both AUD and DUD. More than three-quarters of the patients (177 [89.5%]) were managed as inpatients, while the rest were managed as outpatients.

### 3.3. Direct Treatment Costs of Substance Use Disorder

The direct treatment cost of SUD comprises the cost of medicines, laboratory tests, professional health consultations, psychosocial therapy, behavioral therapy, inpatient admission, and medical devices. Different classes of medicines, including antipsychotics, antidepressants, anticonvulsants, mood stabilizers, antibacterial, and analgesics, were employed to treat SUDs. The average costs of treatment for alcohol use disorder, drug use disorder, and drug and alcohol used disorder were ₦146,425.38 ± 573,88.84, ₦135,282.09 ± 53,190.39, and ₦143,877.33 ± 68,662.04, respectively. This translates to $384.82, $355.53, and $378.12, respectively. The highest contributors to SUD treatment cost are inpatient admissions and the cost of medicines; inpatient admissions include the cost of accommodation, feeding, and laundry. Medical devices had the lowest cost (Table 2). The average cost incurred for the different types of medicines used for the management of SUD in our study population is shown in Table 3. Sedatives and antidepressants had the highest costs.

A comparison of the total mean cost of treatment across demographic characteristics of the patients showed a significant difference in the cost of SUD treatment across the age (years) groups (*F* value = 2.375; *p* < 0.05). Patients below 30 years spent less than ₦140,000 on an average for SUD treatment, while patients who are 30 years and above spent more than ₦140,000 within the 6 months which we assessed. A significant difference was also seen between inpatients and outpatients on the cost of SUD treatment at *t* = 5.782 (*p* < 0.05). Inpatients spent more with an average treatment cost of ₦147,790.90 compared to outpatients with ₦69,521.68. Compared to patients who reside in Lagos, patients in Ogun state incurred higher costs for SUD treatment (*t* = 4.964; *p* < 0.05). No significant difference was seen in the cost of SUD treatment for gender, marital status, educational qualification, and occupation (Table 4).

Analysis of variance among the different categories of SUDs on the associated direct cost of treatments revealed a significant variation in the total cost of psychosocial or behavioral therapy and the total cost of medical devices (*p*=0.002 and 0.001, respectively; Table 5). However, there was no significant variation among the different categories of SUDs on the associated overall direct treatment cost (*F* value = 0.556).

Further multiple comparisons of the statistically significant variables using a post hoc test indicated that the cost of psychosocial or behavioral therapy is highest in treating DUDs. Medical devices' cost is most increased in treating AUDs.

## 4. Discussion

In this study, the direct medical costs associated with SUD treatment in two tertiary hospitals in South-West, Nigeria, were evaluated. It details a comparison between the cost variations in SUD treatment utilizing a cross-sectional survey of treatment charts of patients managed in the health facilities. This model is in tandem with economic evaluation models of direct costs and cost-effectiveness in varying disease conditions [11]. The results reflect that age, marital status, and occupation were associated with AUD and DUD. Unemployed persons accounted for 36.18% of SUD patients, which was slightly higher than that of students, which accounted for 32.67% of the entire study population. This result is similar to that of White et al. [12], where identified patients at risk for prescription opioid use fell in the category of young unemployed individuals.

The direct cost associated with the SUD treatment for alcohol and drug use was ₦143,877.33 ± 68,662.04, with the highest contributor to this cost being the patients' need for hospitalization. In Nigeria, the minimum monthly salary wage is ₦30,000 ± 982.04 in the federal civil service. Over 60.9% of Nigerians living on less than $1 a day will not afford health care costs associated with SUD [13]. The direct cost of SUD management would be equivalent to 5 months' salary for an individual living on the minimum wage. Only approximately 5% of the Nigerian population is currently enrolled with the Nigerian national health insurance scheme [14, 15]. Also, the impact of the direct cost of SUD is borne mainly by the patients and their caregivers. This finding is not consistent with the situation in other countries such as the United States of America, where health insurance companies bear health care costs. White et al. [12] stated that 60% to 95% of the direct cost was carried by insurance companies, limiting out-of-pocket payments by the caregivers. With the rising rate of inflation, which puts the dollar at 380.5 naira to $1 [16], and a population of 152 million Nigerians living on less than $2 a day (representing about 80% of the country's estimated 190 million people), the average cost of SUD treatment as found in this study is considered a high cost to bear by the country's average citizen. The findings from this study suggest that SUD is associated with an increasingly significant economic and public health burden. Relapse is often a recurring factor in managing substance use disorder, and readmissions occur frequently and almost certainly. Sometimes, caregivers transfer patients to other facilities when relapse occurs. When patients are readmitted due to a relapse, they must undergo all the initial testing and therapies offered in the previous admissions. This further drives up the incurred costs. However, patients who were readmitted after a prior admission were not taken into consideration in our study.

A focus on the age range demography clearly shows increased management cost in patients 30 years and older compared to younger patients. This observation may be due to the presence of other health comorbidities associated with this age group. Psychosocial and behavioral therapy is a critical part of SUD treatment, and the cost associated with this was statistically higher than the cost associated with medical devices and medications. Statistically significant variations among different categories of SUD on the direct cost of psychosocial/behavioral therapy and medical devices significantly increased the direct cost of treatment. SUD patients were 2.5 times likely to pay more for care in both neuropsychiatric facilities evaluated. Behavioral therapy costs drove these costs. These factors contributed to the higher medical costs associated with SUD compared with non-SUD patients. Interventions from donors such as individuals providing free services via medical mission outreaches may help mitigate the cost implications of SUD in regions where access to these free services is made available to the patient [17]. Other sources of funds for health care bills for unemployed individuals include crowdfunding and financial assistance from family and friends. Patients with SUDs are admitted into the psychiatry hospital primarily for SUD management. However, they are also treated for other comorbid conditions like diabetes, cardiovascular disease, usually in consult with the disease consultants. As seen from our data, all these treatments contribute to the cost of SUD management.

Study limitations: this study focused on direct health care costs and did not consider indirect costs accrued from disability or inability to earn a living due to the patient's hospitalization. The caregiver also will experience revenue loss while caring for SUD patients. This indirect cost could account for as high as 5 to 10 times the direct cost [13]. The direct cost evaluated only patients receiving care in government-owned neuropsychiatric hospitals, which provide health care at a fraction of the cost of their private/for-profit-run counterparts [8, 15, 17]. Also, patients on readmission after a relapse were not accounted for in this study.

## 5. Conclusion and Recommendations

In conclusion, substance use disorder is an expensive condition with a substantial economic burden on the patients and caregivers. With the absence of proper health insurance schemes in Nigeria, the direct cost of SUD treatment primarily driven by behavioral therapy, hospitalization, and medical devices cost is almost wholly borne by the patients. Considering that over 60% of the Nigerian population lives below the poverty line, the direct cost of SUD treatment is grossly unaffordable to the patients. Pharmacoeconomic methods would be needed to guide health expenditures and inform health policy regarding SUD.

## Figures and Tables

**Table 1:** Socio-demographic characteristics of the patients.

		Type of SUD			
Variable	Characteristics	Alcohol, *N* (%)	Drug, *N* (%)	Drug and alcohol, *N* (%)	*p* value
Age (years)	10–19 20–29 30–39 40–49 50–59 60 and above	6 (27.3) 8 (36.4) 4 (18.2) 3 (13.6) 1 (4.5) 0 (0.0)	45 (49.5) 34 (37.4) 8 (8.8) 4 (4.4) 0 (0.0) 0 (0.0)	24 (27.9) 38 (44.2) 17 (19.8) 4 (4.7) 2 (2.3) 1 (1.2)	0.004^*∗*^

Sex	Male Female	17 (77.3) 5 (22.7)	75 (82.4) 16 (17.6)	69 (80.2) 17 (19.8)	0.840

Marital status	Married Not married Widowed Divorced/separated	7 (31.8) 14 (63.6) 1 (4.5) 0 (0.0)	5 (5.50) 83 (91.2) 0 (0.0) 3 (3.3)	14 (16.3) 70 (81.4) 1 (1.2) 1 (1.2)	0.008^*∗*^

Educational level	No formal education Primary education Secondary education Tertiary education	0 (0.0) 1 (4.5) 8 (36.4) 13 (59.1)	0 (0.0) 2 (2.2) 28 (30.8) 61 (67.0)	1 (1.2) 2 (2.3) 30 (34.9) 53 (61.6)	0.889

Tribe	Igbo Yoruba Hausa Others	6 (27.30 12 954.5) 1 (4.5) 3 913.60	30 (33.0) 49 (53.8) 3 (3.3) 9 (9.9)	23 926.7) 45 (52.3) 1 (1.2) 17 (19.8)	0.565

Occupation	Trader Civil servant Professional Student Self-employed Unemployed Retired	3 (13.6) 3 (13.6) 1 (4.5) 4 (18.2) 3 (13.6) 6 (27.3) 1 (4.5)	9 9.9) 4 (4.4) 4 (4.4) 29 (31.9) 8 9 (8.8) 36 (39.6) 12 (13.2)	6 (7.0) 4 (4.7) 8 (9.3) 32 (37.2) 2 (2.3) 30 (34.9) 9 (10.5)	0.005^*∗*^

Setting	Inpatient Outpatient	21 (95.5) 1 (4.5)	79 (86.6) 12 (13.2)	77 (89.5) 9 (10.5)	0.497

State of residence	Lagos Ogun total	21 (95.5) 1 (4.5) 22 (100.0)	87 (95.6) 4 (4.4) 91 (100.0)	54 (62.80 32 (37.2) 86 (100.0)	≤0.001^*∗*^

^*∗*^Statistical significance (*p* value < 0.05).

**Table 2:** Direct costs of treatment of substance use disorders^#^.

Type of SUD	Variable	No. of patients	Min. cost (₦)	Max. cost (₦)	Mean cost (₦)	Std. dev.
Alcohol	Cost of medicines	22	9586.00	109,868.00	39,496.81	27,967.44
	Cost of laboratory tests	7	4250.00	15,750.00	12,500.00	3881.04
	Health professional consultation cost	7	10,000.00	13,000.00	10,428.57	1133.89
	Cost of psycho-social and/or behavioral therapy	6	4000.00	33,000.00	25,666.67	10,893.42
	Inpatient admission cost	18	68,000.00	120,000.00	105,500.00	23,931.89
	Cost of medical devices	6	4000.00	8000.00	5333.33	2065.59
	Overall cost	22	11,420.00	235,118.00	146,425.38	57,388.84

Drug	Cost of medicines	89	1740.00	116,684.00	29,047.76	20,592.82
	Cost of laboratory tests	33	2000.00	55,500.00	15,377.27	7951.04
	Health professional consultation cost	33	4000.00	19,500.00	10,145.45	2015.89
	Cost of psycho-social and/or behavioral therapy	32	4000.00	61,000.00	28,218.91	12,701.30
	Inpatient admission cost	78	25,000.00	129,000.00	97,507.69	28,467.61
	Cost of medical devices	26	4000.00	4000.00	4000.00	0.00
	Overall cost	89	1740.00	247,884.00	135,282.09	53,190.39

Drug and alcohol	Cost of medicines	84	2824.00	223,310.00	34,739.56	29,135.64
	Cost of laboratory tests	46	750.00	30,500.00	15,716.30	6573.43
	Health professional consultation cost	43	4000.00	19,000.00	11,325.58	3254.82
	Cost of psycho-social and/or behavioral therapy	41	1500.00	47,000.00	17,841.46	12,066.71
	In-patient admission cost	70	49,000.00	149,000.00	104,657.14	26,528.43
	Cost of medical devices	10	4000.00	4000.00	4000.00	0.00
	Overall cost	84	2824.00	393,310.00	143,877.33	68,662.04

^#^$1 is equivalent to ₦380.5.

**Table 3:** Classes and costs of medicines used for the treatment of substance use disorders^#^.

Type of SUD	Class of medicine	No. of patients	Min. cost (₦)	Max. cost (₦)	Mean cost (₦)	Std. dev.
Alcohol	Antipsychotics	4	14,122.00	29,810.00	22,919.75	8076.53
	Antidepressants	4	9586.00	60,236.00	30,538.75	22,903.36
	Anticonvulsant	1	47,727.00	47,727.00	47,727.00	0.00
	Mood stabilizers	4	14,571.00	82,800.00	48,960.50	35,661.65
	Sedatives	1	55,467.00	55,467.00	55,467.00	0.00
	Antibacterial	2	32,270.00	67,516.00	49,893.00	24,922.68
	Analgesic	3	11,420.00	109,868.00	50,105.33	52,499.45
	Supplements	1	31,210.00	31,210.00	31,210.00	0.00
	Antihypertensive	2	14,730.00	22,570.00	18,650.00	5543.71

Drug	Antipsychotics	35	1740.00	78,380.00	29,700.00	18,418.64
	Antidepressants	21	9375.00	86,228.00	38,200.75	20,417.96
	Anticonvulsant	18	11,238.00	116,684.00	36,900.80	25,618.22
	Mood stabilizers	7	7198.00	63,928.00	22,700.95	19,824.54
	Sedatives	2	17,595.00	42,235.00	31,900.95	17,423.11
	Antibacterial	5	4884.00	30,413.00	14,840.95	10,676.43
	Supplements	1	29,089.95	29,089.95	29,089.95	0.00

Drug and alcohol	Antipsychotics	24	6205.00	146,030.00	48,750.95	28,339.63
	Antidepressants	17	9637.00	50,130.00	27,651.95	12,348.83
	Anticonvulsant	10	5504.00	223,310.00	49,461.50	62,944.96
	Mood stabilizers	14	9298.00	54,488.00	29,120.50	16,473.98
	Sedatives	8	6930.00	57,622.00	38,527.70	17,704.96
	Antibacterial	5	3500.00	35,605.00	20,297.50	12,028.73
	Analgesic	2	2824.00	31,920.00	17,372.50	20,573.97
	Supplements	1	8610.00	8610.00	8610.50	0.00
	Antihypertensive	3	13,757.00	32,269.00	22,862.95	9259.66

^#^$1 is equivalent to ₦380.5.

**Table 4:** Mean cost of treatment across demographic characteristics of the patients.

		SUD treatment cost (₦)^#^					
Socio-demographic factors	Characteristics	Overall mean cost (₦)	SD	*t*	*p* value	*F*	*p* value
Age (years)	10–19	136,259.7726	52,641.5404			2.375	0.041^*∗*^
	20–29	134,984.8038	71,264.2967				
	30–39	140,309.7241	49,444.1646				
	40–49	182,122.3636	22,918.3269				
	50–59	177,770.3333	42,352.0020				
	60+	263,500.0000	00000.0000				

Sex	Male	140,093.4804	61,530.0442	−0.052	0.959		
	Female	140,661.3684	57,090.0664				

Marital status	Married	155,129.1154	58,187.5060			1.833	0.142
	Not married	137,058.3957	60,576.5892				
	Widowed	218,580.0000	4367.0914				
	Divorced/separated	132,952.0000	64,005.7644				

Educational level	No formal education	00000.0000	00000.0000			0.193	0.825
	Primary education	156,809.2000	25,763.2442				
	Secondary education	139,449.7062	58,883.7906				
	Tertiary education	139,933.5317	62,546.5945				

Tribe	Igbo	139,235.5690	54,319.6168			0.565	0.639
	Yoruba	140,279.8133	59,407.3928				
	Hausa	104,968.5000	107,873.5868				
	Others	146,723.6034	70,357.8963				

Occupation	Trader	139,587.2941	55,709.3954			1.507	0.167
	Civil servant	166,425.1818	32,267.6258				
	Professional	104,268.0000	73,871.0477				
	Student	142,135.8281	62,343.9430				
	Artisan	108,581.8333	110,340.9393				
	Self-employed	152,713.8077	62,557.2211				
	Unemployed	139,859.6306	53,630.8131				
	Retired	221,668.0000	00000.0000				

Setting	Inpatient	147790.9034	54,665.4179	5.782	≤0.001^*∗*^		
	Outpatient	69,521.6842	68,289.5547				

Patient's state of residence	Lagos	130,422.1503	57,657.2282	4.964	≤0.00^*∗*^		
	Ogun	182,237.2973	55,066.3824				

^*∗*^Statistical significance (*p* value < 0.05). ^#^$1 is equivalent to ₦380.5.

**Table 5:** Variation among the different categories of substance use disorders on the direct cost of treatments.

Source of treatment cost	*Df*	*F*	*p* value
Total cost of medicine	2	1.939	0.147
Total cost of laboratory tests	2	0.644	0.528
Total cost of health professional consultation fees	2	1.850	0.164
Total cost of psycho-social/behavioral therapy	2	6.639	0.002^*∗*^
Total cost of inpatient admission	2	1.504	0.225
Total cost of medical devices	2	8.605	0.001^*∗*^
Total cost of treatment of SUD	2	0.556	0.575

^*∗*^Statistical significance (*p* value < 0.05).

## Data Availability

The raw data for the findings of this study are freely available from the corresponding author upon request.
